# Supplementary material for: Tracing the Genetic Evolution of Canine Parvovirus Type 2 (CPV-2) in Thailand
Source: Pathogens. 2022 Dec 2;11(12):1460. doi: 10.3390/pathogens11121460 (PMC9781796; doi:10.3390/pathogens11121460)
Supplement: Supplementary file 1 [file pathogens-11-01460-s001.zip › pathogens-1991050-supplementary.pdf]

Supplementary Table S1. Signalment, date of sample collection, clinical presentation, and vaccination status of the dogs tested positive for CPV-2 isolated from 2018 to 2019.

Dog ID	Breed	Collection date (DD/MM/YYYY)	Age (M)	Vaccination status	Clinical presentation
MUT1	Mixed breed	12/12/2018	12	Unknown	Vomiting and diarrhea
MUT2	Mixed breed	13/12/2018	24	Unknown	Diarrhea
MUT3	Siberian Husky	13/01/2019	6	Unvaccinated	Vomiting and diarrhea
MUT4	German Shepherd	1/3/2019	2	Unvaccinated	Vomiting and diarrhea
MUT5	American Bulldog	13/02/2018	4	Unvaccinated	Vomiting and diarrhea
MUT6	Pomeranian	15/03/2018	2	Unvaccinated	Vomiting and diarrhea
MUT7	Pomeranian	20/04/2018	5	Unvaccinated	Vomiting and diarrhea
MUT8	Mixed breed	19/06/2018	12	Unknown	Diarrhea
MUT9	Poodle	11/3/2019	3	Vaccinated	Diarrhea
MUT10	Mixed breed	1/4/2019	5	Vaccinated	Diarrhea
MUT11	American Bulldog	5/4/2019	4	Unvaccinated	Diarrhea
MUT12	Pomeranian	2/5/2019	3	Vaccinated	Diarrhea
MUT13	Mixed breed	12/5/2019	3	Vaccinated	Diarrhea
MUT14	Pomeranian	22/06/2019	8	Vaccinated	Diarrhea
MUT15	Mixed breed	25/06/2019	3	Vaccinated	Diarrhea

Supplementary Table S2. The selection criteria of CPV-2 representative sequences.

CPV-2a representative sequences (*n* = 40)				
	Origin	Accession #	Year	Inclusion criteria
1	Italy	KX434462	2015	FPV, an out group
2	China	GU569943	1983	A representative sequence of the original CPV-2 circulating before 2000
3	USA	EU659117	1980	A representative sequence of the original CPV-2 circulating before 2000
4	USA	M19296	1979	A representative sequence of the original CPV-2 circulating before 2000
5	Vaccine Nobivac	FJ197846	2007	A representative sequence of prototype virus
6	Vaccine Quantum	GU212792	2009	A representative sequence of prototype virus
7	VaccineVanguardPlus5	MW239610	2018	A representative sequence of prototype virus
8	Vaccine Duramune	FJ222822	N/A	A representative sequence of prototype virus
9	Vaccine Pfizer	FJ197847	2007	A representative sequence of prototype virus
10	USA	AY742953	2003	A representative sequence of new CPV-2a (S297A) circulating in North America between 2000 and 2010
11	USA	EU659118	1981	A representative sequence of CPV-2a circulating in North America before 2000
12	Italy	FJ005259	2008	A representative sequence of CPV-2 circulating in Europe between 2000 and 2010
13	South Korea	EF599096	2005	A representative sequence of CPV-2 circulating in Asia between 2000 and 2010
14	Vietnam	AB054215	1997	A representative sequence of CPV-2 circulating in Southeast Asia before 2000
15	Vietnam	AB054217	1997	A representative sequence of CPV-2 circulating in Southeast Asia before 2000
16	Vietnam	MK357724	2017	A representative sequence of CPV-2 circulating in Southeast Asia after 2015
17	South Korea	EU009200	2006	A representative sequence of CPV-2 circulating in Asia between 2000 and 2010
18	Canada	MF423125	2014	A representative sequence of CPV-2a circulating in North America between 2011 and 2015
19	Italy	MG434745	2017	A representative sequence new CPV-2a (VP2 324Leu) circulating in Europe after 2015
20	Italy	MG434739	2016	A representative sequence new CPV-2a (VP2 324Leu) circulating in Europe after 2015
21	Italy	MG434741	2017	A representative sequence new CPV-2a (VP2 324Leu) circulating in Europe after 2015

22	Italy	FJ005254	2005	A representative sequence of CPV-2 circulating in Europe between 2000 and 2010
23	Brazil	DQ340407	1980	A representative sequence of the CPV-2 circulating in South America before 2000
24	China	DQ354068	2004	A representative sequence of CPV-2 circulating in Asia between 2000 and 2010
25	China	GU569939	2002	A representative sequence of CPV-2 circulating in Asia between 2000 and 2010
26	China	FJ435343	2008	A representative sequence of CPV-2 circulating in Asia between 2000 and 2010
27	China	GU380304	2009	A representative sequence of CPV-2 circulating in Asia between 2000 and 2010
28	China	GU569936	2008	A representative sequence of CPV-2 circulating in Asia between 2000 and 2010
29	South Korea	FJ197825	2007	A representative sequence of CPV-2 circulating in Asia between 2000 and 2010
30	China	JQ686671	2011	A representative sequence of CPV-2 circulating in Asia between 2011 and 2015
31	China	JX660690	2011	A representative sequence of CPV-2 circulating in Asia between 2011 and 2015
32	Uruguay	KM457139	2011	A representative sequence of CPV-2 circulating in South America between 2011 and 2015
33	Uruguay	JF906788	2010	A representative sequence of CPV-2 circulating in South America between 2000 and 2010
34	Uruguay	KM457141	2011	A representative sequence of CPV-2 circulating in South America between 2011 and 2015
35	China	MF467224	2015	A representative sequence of CPV-2 circulating in Asia between 2011 and 2015
36	India	KX469433	2015	A representative sequence of CPV-2 circulating in Asia between 2011 and 2015
37	India	KX219736	2012	A representative sequence of CPV-2 circulating in Asia between 2011 and 2015
38	Singapore	KY083098	2014	A representative sequence of CPV-2 circulating in Southeast Asia between 2011 and 2015
39	Vietnam	MT106238	2017	A representative sequence of CPV-2 circulating in Southeast Asia after 2015
40	Vietnam	LC214970	2013	A representative sequence of CPV-2 circulating in Southeast Asia between 2011 and 2015
CPV-2b representative sequences (n = 34)				
	Origin	Accession #	Year	Inclusion criteria
1	VaccineDuramune	FJ222822	N/A	A representative sequence of prototype virus
2	USA	JX475261	2010	A representative sequence of CPV-2b circulating in North America between 2000 and 2010

3	Italy	FJ005263	2005	A representative sequence of CPV-2b circulating in Europe between 2000 and 2010
4	Germany	FJ005260	1997	A representative sequence of CPV-2b circulating in Europe before 2000
5	Brazil	MF177251	2013	A representative sequence of CPV-2b circulating in South America between 2011 and 2015
6	Brazil	EU659120	1998	A representative sequence of CPV-2b circulating in South America before 2000
7	Ecuador	MF177280	2011	A representative sequence of CPV-2b circulating in South America between 2011 and 2015
8	Portugal	KR559895	2013	A representative sequence of CPV-2b circulating in Europe between 2011 and 2015
9	Vietnam	AB120723	2002	A representative sequence of CPV-2b circulating in Southeast Asia between 2000 and 2010
10	Vietnam	AB054219	1997	A representative sequence of CPV-2b circulating in Southeast Asia before 2000
11	Vietnam	AB054220	1997	A representative sequence of CPV-2b circulating in Southeast Asia before 2000
12	USA	EU659121	1998	A representative sequence of new CPV-2b (S297A) circulating in North America before 2000
13	India	KX469430	2010	A representative sequence of CPV-2b circulating in Asia between 2000 and 2010
14	Vietnam	AB120724	2002	A representative sequence of CPV-2b circulating in Southeast Asia between 2000 and 2010
15	India	KX425921	2010	A representative sequence of new CPV-2b (S297) circulating in Asia between 2000 and 2010
16	Taiwan	KU244254	2015	A representative sequence of CPV-2b circulating in Asia between 2011 and 2015
17	Taiwan	JX048607	2011	A representative sequence of CPV-2b circulating in Asia between 2011 and 2015
18	China	KY937668	2016	A representative sequence of CPV-2b circulating in Asia after 2015
19	China	KR611492	2014	A representative sequence of CPV-2b circulating in Asia between 2011 and 2015
20	China	JQ743891	2010	A representative sequence of CPV-2b circulating in Asia between 2011 and 2015
21	China	KY937657	2016	A representative sequence of CPV-2b circulating in Asia after 2015
22	China	JQ268284	2011	A representative sequence of new CPV-2b circulating in Asia between 2011 and 2015
23	Italy	KX434462	2015	FPV, an out group
24	China	GU569943	1983	A representative sequence of CPV-2b circulating in Asia before 2000
25	USA	EU659117	1980	A representative sequence of prototype virus circulating before 2000

26	USA	M19296	1979	A representative sequence of prototype virus circulating before 2000
27	VaccineNobivac	FJ197846	2007	A representative sequence of prototype virus
28	VaccineQuantum	GU212792	2009	A representative sequence of prototype virus
29	VaccineVanguardPlus5	MW239610	2018	A representative sequence of prototype virus
30	VaccinePfizer	FJ197847	2007	A representative sequence of prototype virus
31	Italy	MF177226	1999	A representative sequence of CPV-2b circulating in Europe before 2000
32	Argentina	JF414817	2003	A representative sequence of CPV-2b circulating in South America between 2000 and 2010
33	Chile	MT585713	2019	A representative sequence of CPV-2b circulating in South America after 2015
34	Vietnam	AB054221	1997	A representative sequence of CPV-2b circulating in Southeast Asia before 2000

CPV-2c representative sequences (*n* = 29)				
	Origin	Accession #	Year	Inclusion criteria
1	Italy	KX434462	2015	FPV, an out group
2	China	GU569943	1983	A representative sequence of the original CPV-2 circulating in Asia before 2000
3	USA	EU659117	1980	A representative sequence of prototype virus circulating before 2000
4	USA	M19296	1979	A representative sequence of prototype virus circulating before 2000
5	VaccineNobivac	FJ197846	2007	A representative sequence of prototype virus
6	VaccineQuantum	GU212792	2009	A representative sequence of prototype virus
7	VaccineVanguardPlus5	MW239610	2018	A representative sequence of prototype virus
8	VaccineDuramune	FJ222822	N/A	A representative sequence of prototype virus
9	VaccinePfizer	FJ197847	2007	A representative sequence of prototype virus
10	Italy	FJ222821	2000	A representative sequence of the original CPV-2c (Western clade)
11	Italy	MF177239	2001	A representative sequence of CPV-2c (Western clade) circulating between 2000 and 2010
12	Italy	KX434458	2011	A representative sequence of CPV-2c (Western clade) circulating between 2011 and 2015
13	Australia	KU508692	2015	A representative sequence of CPV-2c (Western clade) circulating between 2011 and 2015
14	Spain	FJ005214	2006	A representative sequence of CPV-2c (Western clade) circulating between 2000 and 2010

15	France	DQ025951	2008	A representative sequence of CPV-2c (Western clade) circulating between 2000 and 2010
16	Germany	FJ005196	1997	A representative sequence of CPV-2c (Western clade) circulating before 2000
17	Argentina	MF177243	2008	A representative sequence of CPV-2c (Western clade) circulating between 2000 and 2010
18	Uruguay	KM457117	2009	A representative sequence of CPV-2c (Western clade) circulating between 2000 and 2010
19	Brazil	MK344453	2015	A representative sequence of CPV-2c (Western clade) circulating between 2010 2015
20	Vietnam	MW239593	2019	A representative sequence of CPV-2c (Asian clade) circulating in Southeast Asia after 2015
21	Vietnam	MT106228	2017	A representative sequence of CPV-2c (Asian clade) circulating in Southeast Asia after 2015
22	Vietnam	LC216906	2013	A representative sequence of CPV-2c (Asian clade) circulating in Southeast Asia between 2010 2015
23	Vietnam	MW239583	2018	A representative sequence of CPV-2c (Asian clade) circulating in Southeast Asia after 2015
24	Taiwan	MN832850	2018	A representative sequence of CPV-2c (Asian clade) circulating in Asia after 2015
25	Vietnam	MT106231	2017	A representative sequence of CPV-2c (Asian clade) circulating in Southeast Asia after 2015
26	Vietnam	MK357731	2017	A representative sequence of CPV-2c (Asian clade) circulating in Southeast Asia after 2015
27	Vietnam	MK357737	2018	A representative sequence of CPV-2c (Asian clade) circulating in Southeast Asia after 2015
28	Vietnam	MW239585	2019	A representative sequence of CPV-2c (Asian clade) circulating in Southeast Asia after 2015
29	Vietnam	MW239608	2019	A representative sequence of CPV-2c (Asian clade) circulating in Southeast Asia after 2015

Supplementary Table S3. The percentages of Thai CPV-2 variants between 2003 and 2019.

Years	CPV-2 variants (%)		
	CPV-2a	CPV-2b	CPV-2c
2003	50	50	0
2004	88	13	0

2008	57	43	0
2009	100	0	0
2010	48	52	0
2014	0	0	100
2016	52	3	45
2017	43	3	53
2018	41	0	59
2019	0	0	100

Supplementary Table S4. The numbers and percentages of Thai CPV-2a variant based on VP2 amino acid mutation patterns.

CPV-2a mutation pattern		Year						
		2003	2004	2008	2009	2010	2018	2003 to 2018
No mutation	Number	3	7	1	0	0	0	11
	Percentage (%)	100	100	25	0	0	0	21
1 mutation	Number	0	0	0	0	2	0	2
	Percentage (%)	0	0	0	0	7	0	4
2 mutations	Number	0	0	0	0	1	0	1
	Percentage (%)	0	0	0	0	3	0	2
3 mutations	Number	0	0	3	5	25	5	38
	Percentage (%)	0	0	75	100	86	100	71
4 mutations	Number	0	0	0	0	1	0	1
	Percentage (%)	0	0	0	0	3	0	2

Supplementary Table S5. The VP2 amino acid mutation patterns of Thai and representative CPV-2a variants.

Thai CPV-2a sequences (n=53)								
	Origin	Accession #	Year	Mutation Pattern	80	267	324	440
1	Thailand	FJ869134	2003	No	R	F	Y	T
2	Thailand	FJ869137	2003	No	R	F	Y	T
3	Thailand	FJ869138	2003	No	R	F	Y	T
4	Thailand	FJ869127	2004	No	R	F	Y	T
5	Thailand	FJ869128	2004	No	R	F	Y	T
6	Thailand	FJ869129	2004	No	R	F	Y	T
7	Thailand	FJ869130	2004	No	R	F	Y	T
8	Thailand	FJ869131	2004	No	R	F	Y	T
9	Thailand	FJ869132	2004	No	R	F	Y	T
10	Thailand	FJ869133	2004	No	R	F	Y	T
11	Thailand	FJ869126	2008	No	R	F	Y	T

12	Thailand	KP715660	2010	Single	R	F	I	T
13	Thailand	KP715662	2010	Single	R	F	I	T
14	Thailand	KP715663	2010	Two	R	F	I	A
15	Thailand	GQ379042	2008	Three	R	Y	I	A
16	Thailand	GQ379043	2008	Three	R	Y	I	A
17	Thailand	GQ379049	2008	Three	R	Y	I	A
18	Thailand	GQ379044	2009	Three	R	Y	I	A
19	Thailand	GQ379045	2009	Three	R	Y	I	A
20	Thailand	GQ379046	2009	Three	R	Y	I	A
21	Thailand	GQ379047	2009	Three	R	Y	I	A
22	Thailand	GQ379048	2009	Three	R	Y	I	A
23	Thailand	KP715679	2010	Three	R	Y	I	A
24	Thailand	KP715658	2010	Three	R	Y	I	A
25	Thailand	KP715659	2010	Three	R	Y	I	A
26	Thailand	KP715661	2010	Three	R	Y	I	A
27	Thailand	KP715664	2010	Three	R	Y	I	A
28	Thailand	KP715665	2010	Three	R	Y	I	A
29	Thailand	KP715666	2010	Three	R	Y	I	A
30	Thailand	KP715667	2010	Three	R	Y	I	A
31	Thailand	KP715668	2010	Three	R	Y	I	A
32	Thailand	KP715669	2010	Three	R	Y	I	A
33	Thailand	KP715670	2010	Three	R	Y	I	A
34	Thailand	KP715671	2010	Three	R	Y	I	A
35	Thailand	KP715673	2010	Three	R	Y	I	A
36	Thailand	KP715674	2010	Three	R	Y	I	A
37	Thailand	KP715675	2010	Three	R	Y	I	A
38	Thailand	KP715676	2010	Three	R	Y	I	A
39	Thailand	KP715677	2010	Three	R	Y	I	A
40	Thailand	KP715678	2010	Three	R	Y	I	A
41	Thailand	KP715680	2010	Three	R	Y	I	A
42	Thailand	KP715681	2010	Three	R	Y	I	A
43	Thailand	KP715682	2010	Three	R	Y	I	A
44	Thailand	KP715683	2010	Three	R	Y	I	A
45	Thailand	KP715684	2010	Three	R	Y	I	A
46	Thailand	KP715685	2010	Three	R	Y	I	A
47	Thailand	KP715686	2010	Three	R	Y	I	A
48	Thailand	MN270938	2018	Three	R	Y	I	A
49	Thailand	MN270939	2018	Three	R	Y	I	A
50	Thailand	MN270940	2018	Three	R	Y	I	A
51	Thailand	MN270941	2018	Three	R	Y	I	A
52	Thailand	MN270942	2018	Three	R	Y	I	A

53	Thailand	KP715672	2010	Four	T	Y	I	A
CPV-2a representative sequences (n=40)								
	Origin	Accession #	Year	Mutation Pattern	80	267	324	440
1	Italy	KX434462	2015	N/A (FPV)	K	F	Y	T
2	China	GU569943	1983	No	R	F	Y	T
3	USA	EU659117	1980	No	R	F	Y	T
4	USA	M19296	1979	No	R	F	Y	T
5	VaccineNobivac	FJ197846	2007	No	R	F	Y	T
6	VaccineQuantum	GU212792	2009	No	R	F	Y	T
7	VaccineVanguardPlus5	MW239610	2018	No	R	F	Y	T
8	VaccineDuramune	FJ222822	N/A	No	R	F	Y	T
9	VaccinePfizer	FJ197847	2007	No	R	F	Y	T
10	USA	AY742953	2003	No	R	F	Y	T
11	USA	EU659118	1981	No	R	F	Y	T
12	Italy	FJ005259	2008	No	R	F	Y	T
13	South Korea	EF599096	2005	No	R	F	Y	T
14	Vietnam	AB054215	1997	No	R	F	Y	T
15	Vietnam	AB054217	1997	No	R	F	Y	T
16	Vietnam	MK357724	2017	No	R	F	Y	T
17	South Korea	EU009200	2006	Single	R	F	Y	A
18	Canada	MF423125	2014	Single	R	F	I	T
19	Italy	MG434745	2017	Single	R	F	I	T
20	Italy	MG434739	2016	Single	R	F	I	T
21	Italy	MG434741	2017	Single	R	F	I	T
22	Italy	FJ005254	2005	Single	R	F	Y	A
23	Brazil	DQ340407	1980	Single	R	F	I	T
24	China	DQ354068	2004	Single	R	F	I	T
25	China	GU569939	2002	Single	R	F	I	T
26	China	FJ435343	2008	Single	R	F	I	T
27	China	GU380304	2009	Single	R	F	I	T
28	China	GU569936	2008	Single	R	F	I	T
29	South Korea	FJ197825	2007	Single	R	F	Y	A
30	China	JQ686671	2011	Three	R	Y	I	A
31	China	JX660690	2011	Three	R	Y	I	A
32	Uruguay	KM457139	2011	Three	R	Y	I	A
33	Uruguay	JF906788	2010	Three	R	Y	I	A
34	Uruguay	KM457141	2011	Three	R	Y	I	A
35	China	MF467224	2015	Three	R	Y	I	A
36	India	KX469433	2015	Three	R	Y	I	A
37	India	KX219736	2012	Three	R	Y	I	A
38	Singapore	KY083098	2014	Three	R	Y	I	A

39	Vietnam	MT106238	2017	Three	R	Y	I	A
40	Vietnam	LC214970	2013	Three	R	Y	I	A

Supplementary Table S6. The VP2 nucleotide mutations of 2018 Thai CPV-2a isolates.

Amino acid position		80			267	324		440	
Nucleotide position		238	239	240	800	970	971	1318	1320
Reference nucleotide		A	A	A	T	T	A	A	A
1	CPV2a/Thailand/MN270938/2018	C	G	T	A	A	T	G	G
2	CPV2a/Thailand/MN270939/2018	C	G	T	A	A	T	G	G
3	CPV2a/Thailand/MN270940/2018	C	G	T	A	A	T	G	G
4	CPV2a/Thailand/MN270941/2018	C	G	T	A	A	T	G	G
5	CPV2a/Thailand/MN270942/2018	C	G	T	A	A	T	G	G

Supplementary Table S7. The numbers and percentages of Thai CPV-2b variant based on VP2 amino acid mutation patterns.

CPV-2b mutation pattern		Year				
		2003	2004	2008	2010	2003 to 2009
No mutation	Number	3	1	3	0	7
	Percentage (%)	100	100	100	0	18
1 mutation	Number	0	0	0	1	1
	Percentage (%)	0	0	0	3	3
2 mutations	Number	0	0	0	27	27
	Percentage (%)	0	0	0	87	71
3 mutations	Number	0	0	0	3	3
	Percentage (%)	0	0	0	10	8

Supplementary Table S8. The VP2 amino acid mutation patterns of Thai and representative CPV-2b variants.

Thai CPV-2b sequences (n=38)									
Origin		Accession #	Year	Original CPV-2b	Mutation Pattern	267	297	324	440
1	Thailand	FJ869135	2003	No	No	F	A	Y	T
2	Thailand	FJ869139	2003	No	No	F	A	Y	T
3	Thailand	FJ869165	2003	No	No	F	A	Y	T
4	Thailand	FJ869125	2004	No	No	F	A	Y	T
5	Thailand	FJ869122	2008	No	No	F	A	Y	T
6	Thailand	FJ869123	2008	No	No	F	A	Y	T
7	Thailand	FJ869124	2008	No	No	F	A	Y	T
8	Thailand	KP715695	2010	No	Single	Y	A	Y	T

9	Thailand	KP715687	2010	No	Two	Y	A	I	T
10	Thailand	KP715688	2010	No	Two	Y	A	I	T
11	Thailand	KP715689	2010	No	Two	Y	A	I	T
12	Thailand	KP715690	2010	No	Two	Y	A	I	T
13	Thailand	KP715691	2010	No	Two	Y	A	I	T
14	Thailand	KP715692	2010	No	Two	Y	A	I	T
15	Thailand	KP715694	2010	No	Two	Y	A	I	T
16	Thailand	KP715696	2010	No	Two	Y	A	I	T
17	Thailand	KP715697	2010	No	Two	Y	A	I	T
18	Thailand	KP715698	2010	No	Two	Y	A	I	T
19	Thailand	KP715699	2010	No	Two	Y	A	I	T
20	Thailand	KP715700	2010	No	Two	Y	A	I	T
21	Thailand	KP715701	2010	No	Two	Y	A	I	T
22	Thailand	KP715702	2010	No	Two	Y	A	I	T
23	Thailand	KP715703	2010	No	Two	Y	A	I	T
24	Thailand	KP715704	2010	No	Two	Y	A	I	T
25	Thailand	KP715705	2010	No	Two	Y	A	I	T
26	Thailand	KP715706	2010	No	Two	Y	A	I	T
27	Thailand	KP715707	2010	No	Two	Y	A	I	T
28	Thailand	KP715709	2010	No	Two	Y	A	I	T
29	Thailand	KP715710	2010	No	Two	Y	A	I	T
30	Thailand	KP715711	2010	No	Two	Y	A	I	T
31	Thailand	KP715712	2010	No	Two	Y	A	I	T
32	Thailand	KP715713	2010	No	Two	Y	A	I	T
33	Thailand	KP715714	2010	No	Two	Y	A	I	T
34	Thailand	KP715715	2010	No	Two	Y	A	I	T
35	Thailand	KP715716	2010	No	Two	Y	A	I	T
36	Thailand	KP715693	2010	No	Three	Y	A	I	A
37	Thailand	KP715708	2010	No	Three	Y	A	I	A
38	Thailand	KP715717	2010	No	Three	Y	A	I	A

CPV-2b representative sequences (n=34)

	Origin	Accession #	Year	Original CPV-2b	Mutation Pattern	267	297	324	440
1	VaccineDuramune	FJ222822	N/A	No	No	F	A	Y	T
2	USA	JX475261	2010	No	No	F	A	Y	T
3	Italy	FJ005263	2005	No	No	F	A	Y	T
4	Germany	FJ005260	1997	No	No	F	A	Y	T
5	Brazil	MF177251	2013	No	No	F	A	Y	T
6	Brazil	EU659120	1998	No	No	F	A	Y	T
7	Ecuador	MF177280	2011	No	No	F	A	Y	T
8	Portugal	KR559895	2013	No	No	F	A	Y	T
9	Vietnam	AB120723	2002	No	No	F	A	Y	T
10	Vietnam	AB054219	1997	No	No	F	A	Y	T

11	Vietnam	AB054220	1997	No	No	F	A	Y	T
12	USA	EU659121	1998	No	No	F	A	Y	T
13	India	KX469430	2010	No	Single	Y	A	Y	T
14	Vietnam	AB120724	2002	No	Single	Y	A	Y	T
15	India	KX425921	2010	No	Single	Y	A	Y	T
16	Taiwan	KU244254	2015	No	Two	Y	A	I	T
17	Taiwan	JX048607	2011	No	Three	Y	A	I	A
18	China	KY937668	2016	No	Three	Y	A	I	A
19	China	KR611492	2014	No	Three	Y	A	I	A
20	China	JQ743891	2010	No	Three	Y	A	I	A
21	China	KY937657	2016	No	Three	Y	A	I	A
22	China	JQ268284	2011	No	Three	Y	A	I	A
23	Italy	KX434462	2015	N/A (FPV)	No	F	S	Y	T
24	China	GU569943	1983	Yes	No	F	S	Y	T
25	USA	EU659117	1980	Yes	No	F	S	Y	T
26	USA	M19296	1979	Yes	No	F	S	Y	T
27	VaccineNobivac	FJ197846	2007	Yes	No	F	S	Y	T
28	VaccineQuantum	GU212792	2009	Yes	No	F	S	Y	T
29	VaccineVanguardPlus5	MW239610	2018	Yes	No	F	S	Y	T
30	VaccinePfizer	FJ197847	2007	Yes	No	F	S	Y	T
31	Italy	MF177226	1999	Yes	No	F	S	Y	T
32	Argentina	JF414817	2003	N/A	No	F	N	Y	T
33	Chile	MT585713	2019	N/A	Single	F	N	I	T
34	Vietnam	AB054221	1997	No	Single	F	A	Y	A

Supplementary Table S9. The numbers and percentages of CPV-2c variants based on VP2 amino acid mutation patterns.

CPV-2c mutation pattern		Year			
		2016	2018	2019	2016 to 2019
No mutation	Number	0	19	0	19
	Percentage (%)	0	100	0	65
1 mutation	Number	2	1	1	4
	Percentage (%)	86	5	9	14
2 mutations	Number	0	2	4	6
	Percentage (%)	0	20	80	21

Supplementary Table S10. The VP2 amino acid mutation patterns of Thai CPV-2c and representative CPV-2c variants.

Thai CPV-2c sequences (n=29)					
Origin	Accession #	Year	Mutation Pattern	5	447

1	Thailand	MN270943	2018	No	A	I
2	Thailand	MN270944	2018	No	A	I
3	Thailand	MN270945	2018	No	A	I
4	Thailand	MN270946	2018	No	A	I
5	Thailand	MN270947	2018	No	A	I
6	Thailand	MN270948	2018	No	A	I
7	Thailand	MN270949	2018	No	A	I
8	Thailand	MN270950	2018	No	A	I
9	Thailand	MN270951	2018	No	A	I
10	Thailand	MN270952	2018	No	A	I
11	Thailand	MN270953	2018	No	A	I
12	Thailand	MN270954	2018	No	A	I
13	Thailand	MN270955	2018	No	A	I
14	Thailand	MN270956	2018	No	A	I
15	Thailand	MN270957	2018	No	A	I
16	Thailand	MN270958	2018	No	A	I
17	Thailand	MN270959	2018	No	A	I
18	Thailand	MN270960	2018	No	A	I
19	Thailand	MN270961	2018	No	A	I
20	Thailand	MH711894	2016	Single	G	I
21	Thailand	MH711902	2016	Single	G	I
22	Thailand	ON323036	2018	Single	G	I
23	Thailand	ON323037	2019	Single	G	I
24	Thailand	ON323038	2018	Two	G	M
25	Thailand	ON323039	2018	Two	G	M
26	Thailand	ON323040	2019	Two	G	M
27	Thailand	ON323041	2019	Two	G	M
28	Thailand	ON323042	2019	Two	G	M
29	Thailand	ON323043	2019	Two	G	M

CPV-2c representative sequences (n=29)						
	Origin	Accession #	Year	Mutation Pattern	5	447
1	Italy	KX434462	2015	N/A (FPV)	A	I
2	China	GU569943	1983	No	A	I
3	USA	EU659117	1980	No	A	I
4	USA	M19296	1979	No	A	I
5	VaccineNobivac	FJ197846	2007	No	A	I
6	VaccineQuantum	GU212792	2009	No	A	I
7	VaccineVanguardPlus5	MW239610	2018	No	A	I
8	VaccineDuramune	FJ222822	N/A	No	A	I
9	VaccinePfizer	FJ197847	2007	No	A	I
10	Italy	FJ222821	2000	No	A	I
11	Italy	MF177239	2001	No	A	I
12	Italy	KX434458	2011	No	A	I

13	Australia	KU508692	2015	No	A	I
14	Spain	FJ005214	2006	No	A	I
15	France	DQ025951	2008	No	A	I
16	Germany	FJ005196	1997	No	A	I
17	Argentina	MF177243	2008	No	A	I
18	Uruguay	KM457117	2009	No	A	I
19	Brazil	MK344453	2015	No	A	I
20	Vietnam	MW239593	2019	Single	G	I
21	Vietnam	MT106228	2017	Single	G	I
22	Vietnam	LC216906	2013	Single	G	I
23	Vietnam	MW239583	2018	Single	G	I
24	Taiwan	MN832850	2018	Single	G	I
25	Vietnam	MT106231	2017	Two	G	M
26	Vietnam	MK357731	2017	Two	G	M
27	Vietnam	MK357737	2018	Two	G	M
28	Vietnam	MW239585	2019	Two	G	M
29	Vietnam	MW239608	2019	Two	G	M
